# Compact Antenna in 3D Configuration for Rectenna Wireless Power Transmission Applications

**DOI:** 10.3390/s21093193

**Published:** 2021-05-04

**Authors:** Alassane Sidibe, Alexandru Takacs, Gaël Loubet, Daniela Dragomirescu

**Affiliations:** 1 Laboratoire d’Analyse et d’Architecture des Systèmes du Centre National de la Recherche Scientifique (LAAS-CNRS), Université de Toulouse, Centre National de la Recherche Scientifique (CNRS), Institut National des Sciences Appliqués de Toulouse (INSA), Université Paul Sabatier, Toulouse III (UPS), 31400 Toulouse, France; alexandru.takacs@laas.fr (A.T.); gael.loubet@laas.fr (G.L.); daniela.dragomirescu@laas.fr (D.D.); 2 Uwinloc, 9 Rue Humbert Tomatis, 31200 Toulouse, France

**Keywords:** compact antenna, wireless power transmission (WPT), energy harvesting, rectenna, wireless sensors

## Abstract

This work presents methods for miniaturizing and characterizing a modified dipole antenna dedicated to the implementation of wireless power transmission systems. The antenna size should respect the planar dimensions of 60 mm × 30 mm to be integrated with small IoT devices such as a Bluetooth Lower Energy Sensing Node. The provided design is based on a folded short-circuited dipole antenna, also named a T-match antenna. Faced with the difficulty of reducing the physical dimensions of the antenna, we propose a 3D configuration by adding vertical metallic arms on the edges of the antenna. The adopted 3D design has an overall size of 56 mm × 32 mm × 10 mm at 868 MHz. Three antenna-feeding techniques were evaluated to characterize this antenna. They consist of soldering a U.FL connector on the input port; vertically connecting a tapered balun to the antenna; and integrating a microstrip transition to the layer of the antenna. The experimental results of the selected feeding techniques show good agreements and the antenna has a maximum gain of +1.54 dBi in the elevation plane (E-plane). In addition, a final modification was operated to the designed antenna to have a more compact structure with a size of 40 mm × 30 mm × 10 mm at 868 MHz. Such modification reduces the radiation surface of the antenna and so the antenna gain and bandwidth. This antenna can achieve a maximum gain of +1.1 dBi in the E-plane. The two antennas proposed in this paper were then associated with a rectifier to perform energy harvesting for powering Bluetooth Low Energy wireless sensors. The measured RF-DC (radiofrequency to direct current) conversion efficiency is 73.88% (first design) and 60.21% (second design) with an illuminating power density of 3.1 µW/cm^2^ at 868 MHz with a 10 kΩ load resistor.

## 1. Introduction

Over the last decades, we have been faced with the miniaturization of electronic devices, especially in the field of wireless systems. The aim is to have multiple functionalities on an ever-smaller surface area. Recent IoT applications (Internet of Things) tend to employ tiny and low power electronic components [1]. However, batteries are still widely used for powering the devices despite their significant size and the frequent need for replacement. An alternative is using a battery-free system powered by energy harvesting (EH) or wireless power transmission (WPT), for instance, based on a rectenna circuit.

A rectenna is a combination of a rectifier and an antenna used to scavenge ambient or specially generated far-field radiofrequency (RF) waves [2]. The implementation of a rectenna in a battery-free system can allow increasing its lifetime, reducing its manufacturing costs, while ensuring reliable performances for decades. Several kinds of application exist, such as, for instance, rectennas, which were developed for a biomedical device pasted on the human body [3] or used in the IoT domain [4,5,6].

The antenna is a ubiquitous element in IoT and other wireless applications. However, the required size stays important due to the important dependence of the geometrical elements with the targeted wavelength. Several miniaturization techniques are described in the literature. Structural modification on a Printed Circuit Board (PCB) antenna consists of acting on the geometry of the antenna or by adding another element on the antenna shape.

In this sense, a coupling element, such as a rectangular ring, can allow a reduction in size and an increase in bandwidth as presented in [7]. Traditionally, small size antennas are designed by using meander lines which reduce the resonant frequency [8]. Fractal geometry is also used to miniaturize antennas [9]. Another possibility is to add reactive loading elements. This technique is employed in [10] through an LC load (a combination of a lumped inductor and a distributed capacitor). Miniaturization can also be achieved by reducing the guided wavelength through the use of a higher permittivity material, such as a ceramic—polymer composite [11]. Recent research activities were focused on developing small antennas on metamaterial as presented in [12]. In this paper, we present a miniaturization technique which consists of shaping the antenna to form a three-dimensional (3D) structure tuned for the Industrial Scientific and Medical (ISM) 868 MHz frequency band. This configuration allows us to have an electrically small antenna as described in Section 2. A more compact antenna based on the previous one is designed to compare the trade-off between size and rectenna performances. Then, Section 3 presents the experimental results of the fabricated antennas with different feeding methods to validate the use of the U.FL connector for the next steps. Simulations were carried out on the Ansys HFSS software and verified with far-field measurements performed in an anechoic chamber. The final section of this paper describes an original concept of powering a Bluetooth Low Energy Sensing Node embedded in concrete element with a compact and efficient rectenna design.

## 2. Design of a Compact 3D Dipole Antenna

### 2.1. Antenna Miniaturization Methods and the First Design of the Compact 3D Dipole

In this study, we proposed a miniaturized antenna design for WPT applications. An antenna is a resonant structure with a proper frequency depending on its length. Therefore, there are size and performance limitations for small antennas [13,14]. The size reduction imposes a smaller radiation resistance, so a lower radiation efficiency and a bandwidth limitation. Wheeler defines a electrically small antenna as one defined by the formula given in Equation (1). It means that the antenna sphere is smaller than the radian sphere, also defined as Wheeler Cap. *λ* is the wavelength at the operating frequency, *k* represents the free-space wavenumber and a is the minimum antenna sphere radius. The choice of proposing an electrically small antenna should allow us to have an antenna design in the maximum planar size of 60 mm × 30 mm. Nevertheless, the antenna bandwidth reduction does not matter with the applications in the ISM 868 MHz frequency band. The targeted antenna gain is about +1 dBi less compared to a conventional dipole antenna gain (+2.15 dBi).

(1) $$k\cdot a<1\;with\;k=\frac{2\pi }{\lambda }$$

**Figure 1:**
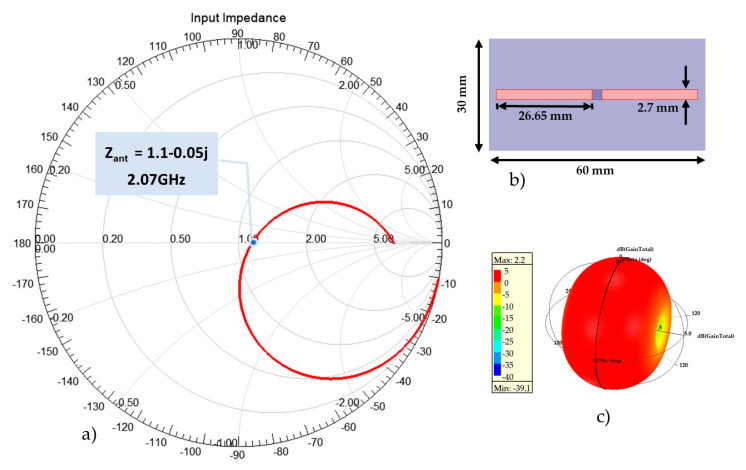
Conventional dipole antenna simulated on the required size dimensions. (**a**) The input impedance representation on Smith chart; (**b**) Half-wavelength antenna dimensions; (**c**) Simulated 3D radiation pattern at 2 GHz.

The proposed design is based on a dipole antenna according to its multiple advantages, such as the symmetry of its shape and radiation pattern, its easy and low-cost fabrication, and the possibility of receiving balanced signals. The required dimensions of 60 mm × 30 mm allow us to have a conventional half-wavelength dipole antenna at 2 GHz, as presented in Figure 1.

**Figure 2:**
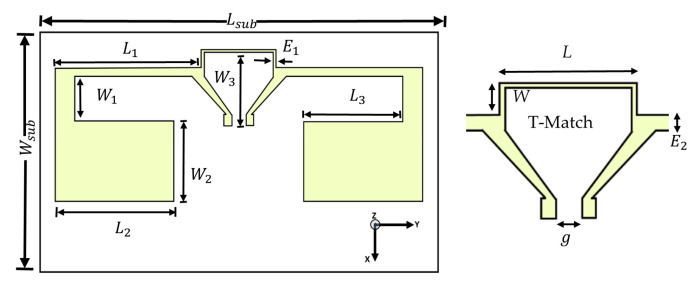
Detailed geometry of the compact, T-match dipole antenna on an FR4 substrate (*L_sub_* = 56 mm, *W_sub_* = 32 mm, *L*_1_ = 20.65 mm, *W*_1_ = 5.95 mm, *L*_2_ = 16.8 mm, *W*_2_ = 10.5 mm, *L*_3_ = 14 mm, *W*_3_ = 9.625 mm, *E*_1_ = 0.35 mm, *E*_2_ = 1.05 mm, g = 2.03 mm).

**Figure 3:**
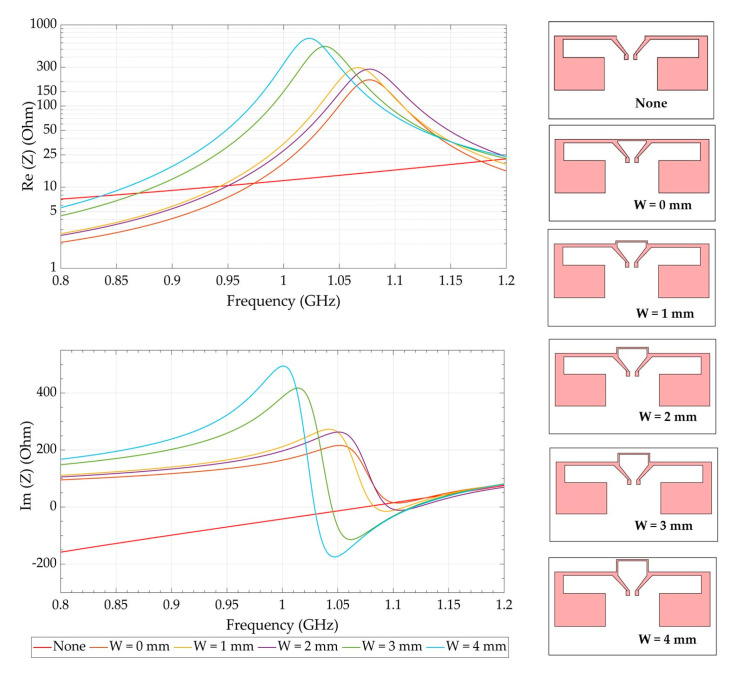
Evolution of the input impedance (Real and Imaginary parts) as function of the frequency with various shorting line width (W) values.

Starting from this antenna at 2 GHz, we had investigated a technique for reducing the physical dimensions of the antenna without significantly degrading its performances (+1 dBi less of the gain). The planar structure of the final antenna design on an FR4 substrate is presented in Figure 2. Overall, a folded dipole antenna designed after size optimization (Antenna in Figure 3 stamped “None”) presents a resonant frequency close to 1.12 GHz regarding the return loss (S11) equal to −5.2 dB.

This antenna can be considered as a half-wavelength dipole antenna at 1.12 GHz. The total length of each folded arm (L_1_ + W_1_ + L_2_ = 43.4 mm) is closed to the quarter wavelength (<0.25·λ_g_ = 31.9 mm). Figure 3 presents the width tuning of the inductive shorting loop to make a T-match structure [15]. The resonance frequency is greatly influenced by the width and the length of the T-match structure. As seen in Figure 3, the absence of the T-match structure (None) shows a significant resonant frequency of the antenna higher than 1.12 GHz where the impedance is (17.35 + j·27.3) Ω. The shorting line (W = 0) connected to the two dipole arms downshifts the resonant frequency around 1.07 GHz. Thereafter, the geometric parameters (W and L) were adjusted to match the input impedance of the antenna (50 Ω) at lower frequency.

**Figure 4:**
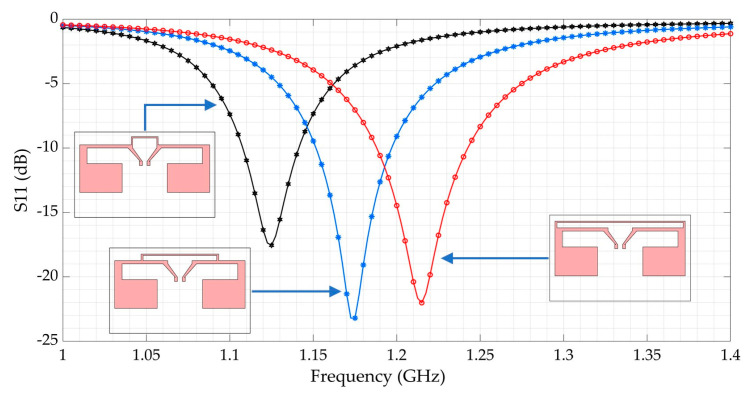
Simulated S11 (return loss) of the antenna for different configurations; L = 10 mm (black), L = 30 mm (blue) and L = 51.4 mm (red).

The simulated results of the optimization on HFSS software are displayed in Figure 3 and Figure 4. The return loss (reflection coefficient S_11_) indicates how much the power is reflected on the input port when the antenna is excited. A practical criterion for the antenna (impedance) input matching is generally specified at −10 dBm at least for a reference impedance of 50 Ω. As represented in Figure 4, the greater the length (L) of the short-circuited line, the more the antenna resonates at higher frequency.

**Figure 5:**
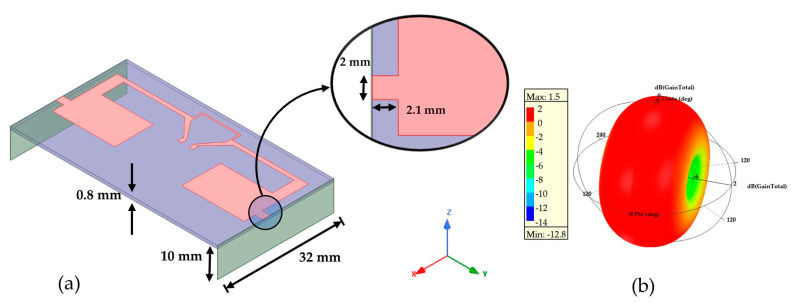
(**a**) Geometry of the 3D configuration antenna with the connected metallic arms; (**b**) Simulated 3D gain polar plot at 868 MHz.

For the next miniaturization step, the T-match structure parameters were fixed to those which give the lower resonant frequency (W = 4 mm and L = 10 mm). To downshift the operating frequency obtained from the planar antenna from 1.12 GHz to 868 MHz, a 3D configuration was investigated. The operating/resonant frequency of the antenna can be downshifted by connecting two metal strands [16]. In our case, two capacitive metallic arms with a height of 10 mm are vertically connected to the planar antenna with a short transmission line (Figure 5a). This line’s position at the edge of the planar antenna was tuned to obtain an operating frequency as close as possible to 868 MHz with a bandwidth of 30.7 MHz. 

In our application at the European ISM 868 MHz frequency band, a narrow band antenna is not critical. The radiation pattern looks like a doughnut shape and the maximum simulated gain is +1.5 dBi (Figure 5b).

### 2.2. Second Miniaturized Antenna Design

**Figure 6:**
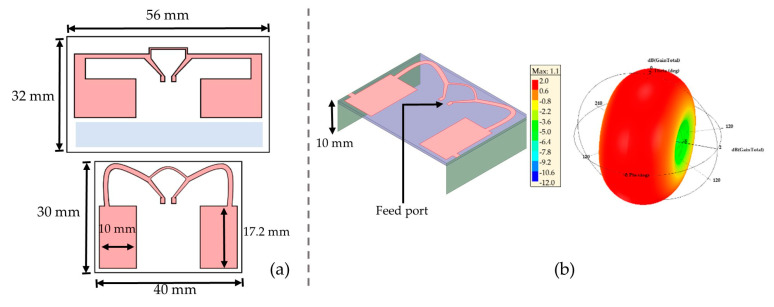
Designed antennas on HFSS: (**a**) The first miniaturized antenna and the second miniaturized antenna named A2: 3D FDA; (**b**) 3D polar plot of the radiation pattern of A2 antenna at the resonant frequency (HFSS results).

In terms of compactness, the previous antenna was modified and two different antennas (A1 and A2) were designed, respectively, the unconnected and connected antenna to the metallic arms. The horizontal arms of the planar dipole are folded in a spline shape to occupy the blue part display in Figure 6a and reduce the length (*L_sub_*) of the planar antenna. By the way, the width of the arms was adjusted to the substrate width. This curving shape was obtained after several optimized simulations, and the size of A2 (represented in Figure 6b) is only 40 mm × 30 mm × 10 mm.

**Figure 7:**
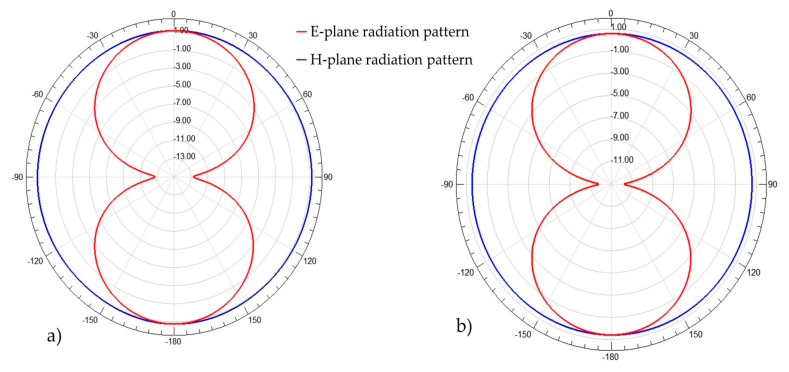
Radiation pattern on the E-plane and H-plane at 868 MHz. (**a**) The antenna first 3D dipole antenna in Section 2.1; (**b**) The modified 3D dipole antenna (A2).

The simulations performed show an operating frequency at 1.15 GHz for A1 and 868 MHz for A2. However, there is a small gain reduction of 0.4 dBi compared to the previous designed antenna, so that we still respect the first condition of a maximum +1 dBi loss on the gain from a conventional dipole antenna. The radiation patterns in the E-plane and the H-plane are presented in Figure 7.

## 3. Characterization of the Designed Antennas

The designed antenna was manufactured in an FR4 substrate with a thickness of 0.8 mm. Dipole antennas require a balanced-unbalanced (balun) circuit or a microstrip transition for a coaxial measurement. The balun allows canceling the flowing current on the outside surface of the outer coaxial conductor and then affects the measurement [17]. In the literature, several dipole antenna-feeding techniques were proposed. A microstrip tapered balun was used as a feeding line in [18] and a microstrip-to-coplanar (CPW) feed network balun for a flexible bowtie antenna in [19]. On the other hand, in many electronic devices especially in IoT, a surface mount coaxial U.FL connector is strongly used for characterization and RF connection for embedded antennas. Its advantages are its low cost, small size and light weight.

**Figure 8:**
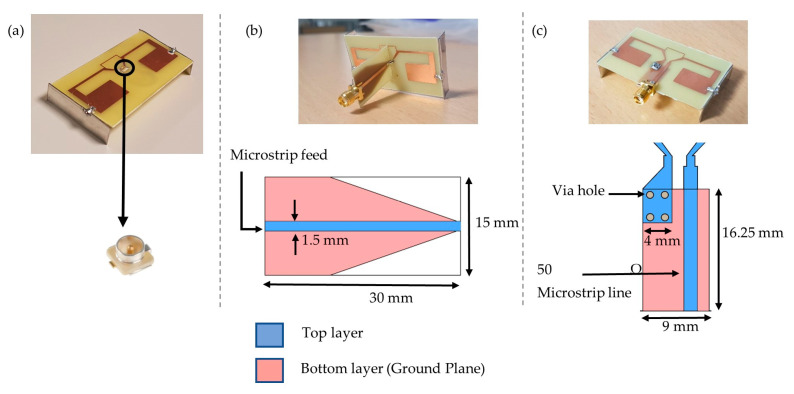
(**a**) Antenna with a U.FL connector named AC; (**b**) Antenna with connected tapered balun named ATB; (**c**) Antenna with integrated microstrip transition named AIT.

**Figure 9:**
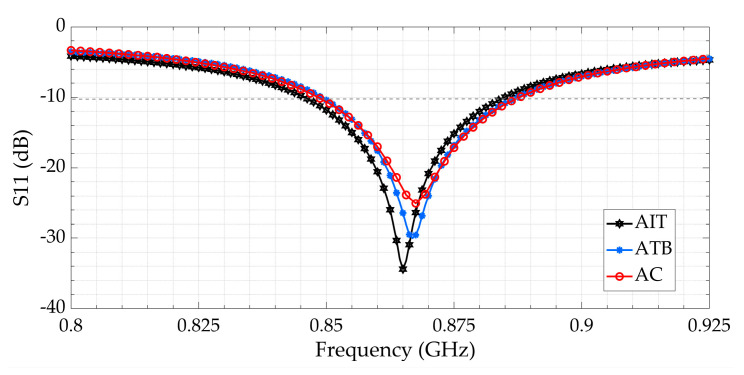
Comparison of the measured return loss (S11) of the D1 antenna with different feeding methods.

**Figure 10:**
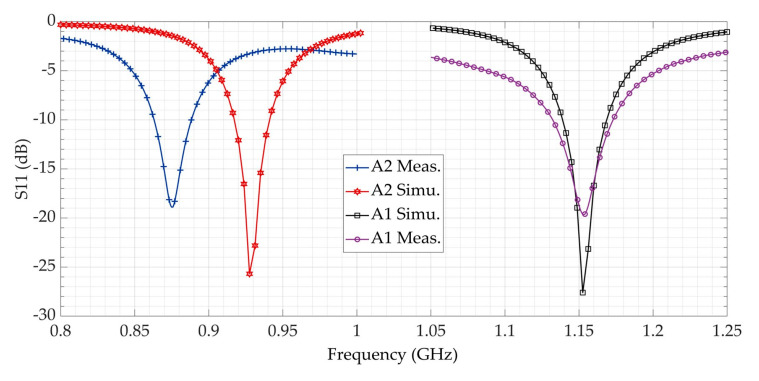
Measured and simulated reflection coefficient of the A1 and A2 antennas.

**Figure 11:**
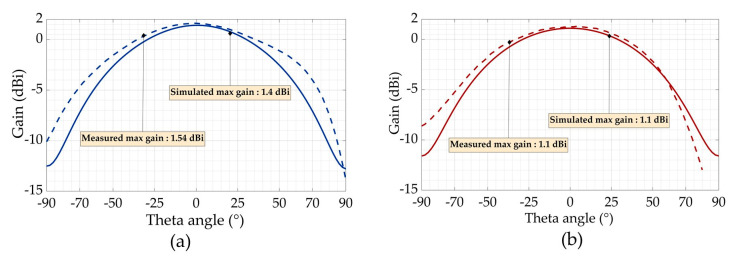
Simulated (dashed line) and measured (continuous line) radiation pattern (gain plot in the E-plane): (**a**) AC antenna; (**b**) A2 antenna at the resonant frequency (868 MHz).

To well characterize our designed antenna, three feeding methods were selected. The first feeding technique carried in this paper is to use a U.FL connector [20]. The pads of the connector were soldered on the antenna feeding pads, as seen in Figure 8a. The second method consists of vertically connecting a designed tapered microstrip balun (Figure 8b). The last one is a microstrip transition: while one pad of the antenna is connected to a 50 Ω transmission line, the other balanced pad is connected to the ground plane (Figure 8c). For the return loss (S_11_) and the gain measurements, a compatible coaxial cable was connected to the vector network analyzer (VNA). The measured return loss is plotted in Figure 9 (by using three different connection methods). They all fit well to each other but present a 15 MHz frequency shift compared to the simulation. The same frequency shift can be observed in Figure 10 between simulation and measurement for the curved 3D antenna A2. Without the two connected arms, the simulated and measured return loss fit perfectly, but once they are connected, a 50 MHz frequency shift appears. The difference is mainly due to the soldering effect and the vertical capacitive arms whose dimensions are not perfectly respected as compared with the simulated ones. The A2 antenna was measured with a U.FL connector and resonates at 878 MHz, as shown in Figure 10. The measured and simulated gain in the E-plane are shown in Figure 11.

Table 1 compares in terms of performances and size some state-of-the-art antennas and the presented solution operating in ISM 868 MHz or ISM 915 MHz frequency bands. The proposed compact 3D antenna was implemented on an FR4 substrate that is lossier as compared with the substrates used by other state-of-the-art designs.

## 4. Rectenna and Wireless Power Transmission Experimentations

The antennas presented in this paper are more compact than the 3D antennas in [16,23], and exhibit the best tradeoff between size, gain and bandwidth and can be good candidates for WPT applications. They were integrated in rectennas used for battery-free wireless sensors embedded in concrete structures [24,25].

**Figure 12:**
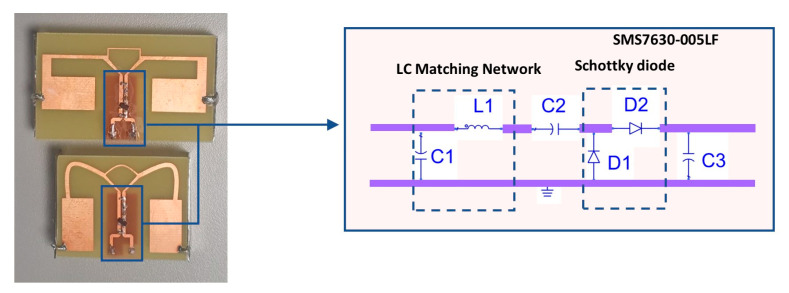
Manufactured rectenna with the rectifier schematic.

The rectifier part is designed using ADS/Momentum software from Keysight. The circuit is designed on a microstrip coupled transmission line allowing differential feeding by the dipole antenna (Figure 12). It is composed of a SMS7630-005LF Schottky diode, an LC matching network (L1-C1) ensuring 50 Ω input impedance at the input of the rectifier and a low pass filter formed by a shunt capacitor (C3) and a resistive load (R1). However, considering the nonlinear behavior of the Schottky diode, the rectifier was designed and optimized for a low input power of −15 dBm and a 10 kΩ resistor to emulate the load (that is the input impedance of the power management unit (PMU)). The simulation result was performed and described in [24]. Two rectennas, R1 and R2, in Figure 12 represent the rectifier with the antenna AC without any connector and A2, respectively. They were made by integrating the rectifier with the antennas on the same substrate. Their characterization was performed in an anechoic chamber at the distance of 1.5 m from the patch-transmitting antenna, and the harvested DC power was measured across a 10 kΩ load through a multimeter. The rectennas R1 and R2 allow a harvested DC voltage of 788 mV and 726 mV for 1 µW/cm^2^.

**Figure 13:**
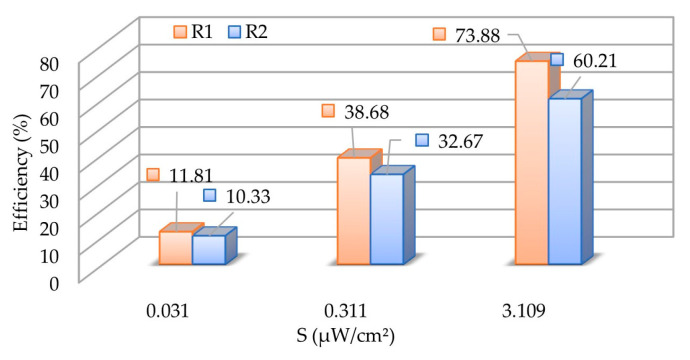
RF-DC conversion efficiency for the rectennas at 868 MHz for various decade power densities.

The conversion efficiency as a function of the power density is presented in Figure 13. It is obtained by computing the formula given in Equation (2), where:

- $P_{DC}$ is the harvested DC power obtained across the load;
- Gt and Gr are the transmitting antenna gain and the receiver antenna gain;
- λ is the wavelength at the operating frequency;
- S is the electromagnetic (EM) power density and $P_{t}$ is the *RF* power from the source;
- d is the distance between the transmitting and receiving antennas.

The variation by decade shows a difference between R1 and R2 of 13.67%, 6.01% and 1.48%. The difference can be explained by the non-linearity of the used Schottky diode. By reducing the planar antenna size of 30%, we reduce the antenna gain of 0.44 dBi and then the RF-DC conversion efficiency of 13% with a power density of 3.1 µW/cm^2^ at 868 MHz. The second rectenna R2 will be preferred in cases where the power density is very low (e.g., 31 nW/cm^2^). For our application, in the aim of supplying the power management unit, which requires a DC voltage of 600 mV at least, R1 was chosen.

(2) $$\eta =100\cdot \frac{P_{DC}}{P_{RF}}=100\cdot \frac{4\cdot \pi \cdot P_{dc}}{S\cdot G_{r}\cdot \lambda^{2}}\;with\;\;S=\frac{E^{2}}{120\cdot \pi }=\frac{30\cdot P_{t}\cdot G_{t}}{d^{2}\cdot 120\cdot \pi }$$

**Figure 14:**
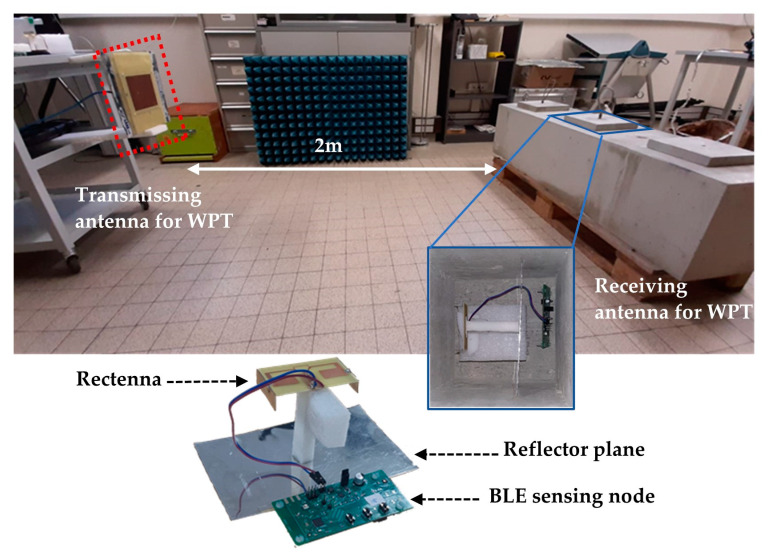
Photograph of the experimental setup for the sensing node embedded and powered by using WPT system in the concrete structure.

In the last set of tests, underrun in our laboratory, an innovative Bluetooth Low Energy (BLE) battery-free wireless sensor network (composed of battery-free sensing nodes and a communicating node [26]) was operated in a harsh environment (Figure 14). Once embedded, the main objectives were to periodically monitor the physical data (e.g., temperature and humidity) of the concrete and broadcast them (by BLE) to the communicating node.

**Figure 15:**
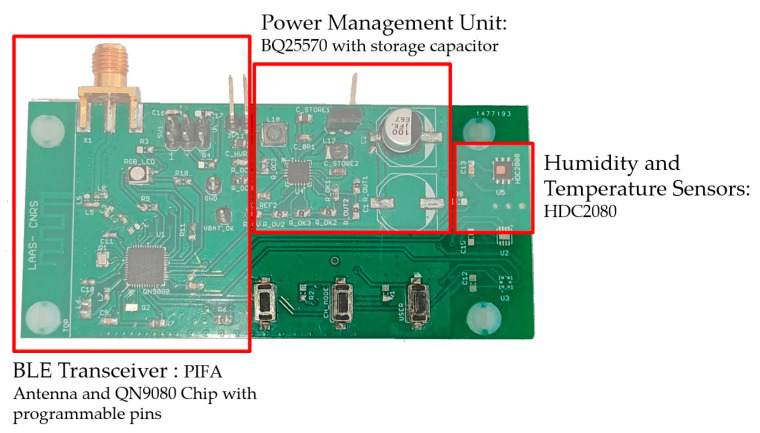
Developed sensing node for BLE communication.

The sensing node in Figure 15 is composed of a rectenna (including the compact 3D antenna), an ultra-low-power BLE System on Chip (SoC) (NXP QN9080) [27], a power management unit microcontroller (Texas Instruments bq25570) [28], a capacitor of 100 µF acting as an energy storage element (Panasonic EEEFK0J101P) [29] and low-power temperature and humidity digital sensors (Texas Instruments HDC2080) [30]. The capacitor was chosen after evaluating the power needed by the PMU (870 µJ) to startup from deep-sleep mode. Through a far field WPT system, the illuminating power over a distance of two meters can be harvested by the rectenna and allow powering the sensing node. Initially empty, the capacitor is charging through the PMU on deep-sleep mode up to 1.5 V and switches on fast charging until the stored voltage reaches 5.3 V. Once it has enough energy stored by the capacitor, it discharges and allows powering the active component on the board (sensors and BLE chip) for a measurement and data transmission.

Preliminary tests, with an EIRP power of +33 dBm, allow a periodicity of charging, measuring and wireless communication equal to at most 190 s, representing the first charge duration (cold start + fast charging + data measurement and transmission).

## 5. Conclusions

An electrically small antenna operating in the ISM 868 MHz frequency band and dedicated to wireless power transmission applications is proposed in this paper. Its design is based on a folded dipole antenna with a shorting line to form a T-match antenna. A 3D configuration allows the exploitation of the Z-plane in the final implementation. Thus, the connection of two metallic arms was operated by the planar antenna to downshift the operating frequency from 1.09 GHz to 868 MHz. The design was modified to apply a spline shape to the planar antenna arms in order to reduce the overall dimensions (in the horizontal plane) from 56 mm × 32 mm to 40 mm × 30 mm.

For the characterization, three feeding methods were used to provide accurate experimental results. In this application, the U.FL connector can be used for measurement but may have limitations at higher frequencies. The experimental results confirm that the proposed 3D antenna exhibits a gain of +1.54 dBi and +1.1 dBi at the operating frequency, respectively, for AC and A2. As shown in Table 1, a tradeoff between the compactness, the maximal gain and the bandwidth should be performed before the design. Our solution is also the most compact and has a maximum gain higher than +1 dBi at the price of a reduced bandwidth. The combination of these antennas and an optimized rectifier performs a high efficiency of 73.88% and 60.31%, respectively, for R1 and R2 with an illuminating power density of 3.1 µW/cm^2^. Future works with a WPT system associated with a battery-free wireless sensor network using BLE are in progress.

## Figures and Tables

**Table 1:** Comparison with the different compact antennas for rectenna of IoT devices in the state of the art.

Ref.	Freq. (MHz)	Type	Max Gain (dBi)	BW (MHz)	Substrate	Size (mm × mm × mm)
[21]	878	Dual Band PIFA	+1.8–+1.9	80 (855–937)	Duroid 5880	80 × 45 (0.03·λ^2^)
[22]	868	UCA PIFA	+0.71	23 (857–880)	FR4	34 × 80 (0.02·λ^2^)
[16]	915	Slot loaded DB folded dipole	+1.87	Not available	Arlon 25N	60 × 60 × 60 (0.006·λ^3^)
[23]	868	3D single arm bowtie	+0.19	90	Ultralam	50 × 50 × 10 (0.0006·λ^3^)
This work	868	3D modified T-match dipole	+1.54	32 (865–897)	FR4	56 × 32 × 10 (0.0004·λ^3^)
			+1.1	26 (862–888)		40 × 30 × 10 (0.0003·λ^3^)

BW: Bandwidth, PIFA: Planar Inverted F-Antenna, DB: Dual Band.
